# Unmasking the Mystery of Renal Neoplasm in a Perimenopausal Woman: A Case Report

**DOI:** 10.7759/cureus.56970

**Published:** 2024-03-26

**Authors:** Arpana Dharwadkar, Charusheela Gore, Gayatri Bhuibhar, Vidya Viswanathan

**Affiliations:** 1 Pathology, Dr. D. Y. Patil Medical College, Hospital & Research Centre, Dr. D. Y. Patil Vidyapeeth (Deemed to be University) Pimpri, Pune, IND

**Keywords:** immunohistochemistry, laproscopic nephrectomy, mixed epithelial and stromal tumor, perimenopausal woman, renal neoplasm

## Abstract

Mixed epithelial and stromal tumor (MEST) is a benign, complex, and rarely encountered renal neoplasm. This case involves a 46-year-old perimenopausal woman who presented with symptoms, such as abdominal pain, burning sensation during urination, increased urinary frequency, and hesitancy. Computed tomography (CT) urography revealed an exophytic, heterogeneously hyperdense mass originating from the interpolar and lower pole parenchyma of the left kidney, suggesting a neoplastic origin. Due to concerns about malignancy and the presence of local symptoms, a laparoscopic-assisted left radical nephrectomy was performed. Histopathological examination of the excised tissue revealed a biphasic neoplasm consisting of epithelial and stromal elements. The epithelial component exhibited cysts and glands of variable sizes, lined by columnar cells and surrounded by stromal tissue. The diagnosis of MESTs of the kidney was established and confirmed through immunohistochemistry. This unique type of benign kidney tumor can be effectively managed through conservative surgery and is associated with a favorable prognosis.

## Introduction

Mixed epithelial and stromal tumor (MEST) is a rare, benign, and intricate renal neoplasm characterized by a biphasic composition seen primarily in adults [1]. It exhibits a complex architecture with both solid and cystic elements, comprised of dilated tubules and cysts lined by cuboidal to columnar epithelium, frequently presenting a distinctive "hobnail" appearance on the surface [2]. Among all renal cancers, MEST accounts for only 0.2% [3]. While the majority of cases exhibit a benign clinical course with favorable outcomes, there is a potential for malignant transformation, as documented in isolated cases in the literature [4]. Initially referred to as congenital mesoblastic nephroma in 1973 by Block et al., the term "mixed epithelial and stromal tumor of the kidney" (MESTK) was coined by Michal and Syrucek in 1998 [5,6].

Currently, MEST is categorized within the MEST family of adult tumors, which ranges from predominantly cystic tumors, like adult cystic nephromas to tumors displaying variable solid and cystic features, as observed in MEST. Microscopically, MEST appears as a multiloculated cystic renal mass with variable solid and cystic components, often showing internal septa [7]. This case report brings attention to the identification of MEST in the kidney of a 46-year-old patient.

## Case presentation

A 46-year-old perimenopausal woman presented with a 15-day history of abdominal pain, burning sensation during urination, increased frequency, and hesitancy. In addition, the patient experienced fever with chills for the past 10 days. The general examination did not reveal any notable findings, and the patient had no significant past or family medical history. Laboratory investigations were done (Table 1). Urine analysis showed the presence of red blood cells (RBCs) at a concentration of 30-50/high-power field, but urine culture results were negative. The serum creatinine level was within the normal range at 0.85 mg/dl.

**Table 1:** Laboratory investigation results

Parameter	Observed value	Normal value
Hemoglobin	10.2 g/dl	11.6-15.0 g/dL
Platelet count	2,80,000 /uL	1,50,000-4,10,000/uL
Leukocyte count	12200/uL	4000-10000/uL

**Figure 1:**
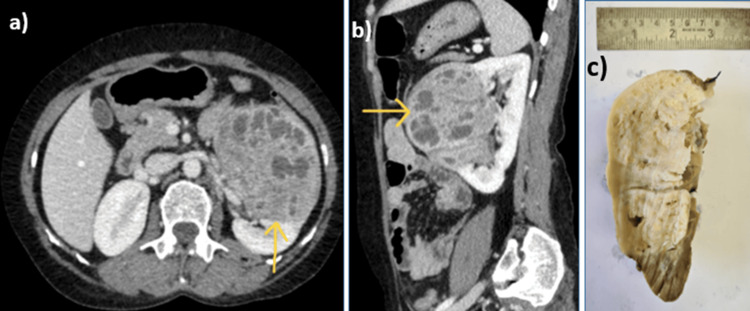
Axial and sagittal CT (a, b) and gross findings of the left kidney (c) Axial and sagittal CT views showing a lesion in the interpolar and upper pole of the kidney (a, b). Gross findings of the left kidney showing multiple cystic and solid areas in the interpolar and upper pole of the kidney (c).

Upon systemic examination, a soft to firm, non-tender mass with a smooth surface, indistinct borders, and ballotable characteristics was palpable in the left hypochondrium. Computed tomography (CT) urography revealed an exophytic, heterogeneously hyperdense lesion measuring 71 x 66 mm. The lesion originated from the interpolar and lower pole parenchyma of the left kidney, accompanied by an inferolateral nodular perinephric extension, indicative of a neoplastic origin. The imaging also revealed tiny calcifications, heterogeneous enhancement during arterial and venous phases, and non-enhancing cystic areas (Figure 1a, 1b). Due to concerns about malignancy and the presence of local symptoms, a laparoscopic-assisted left radical nephrectomy was performed. The gross pathological examination revealed an 8 x 7 x 6 cm, grey-yellow, circumscribed, large, lobulated lesion in the interpolar and upper pole of the kidney. The tumor exhibited multiple solid and cystic areas without necrosis and hemorrhage. Various cystic areas, ranging in size from 2 mm to 2 cm, were observed (Figure 1c).

**Figure 2:**
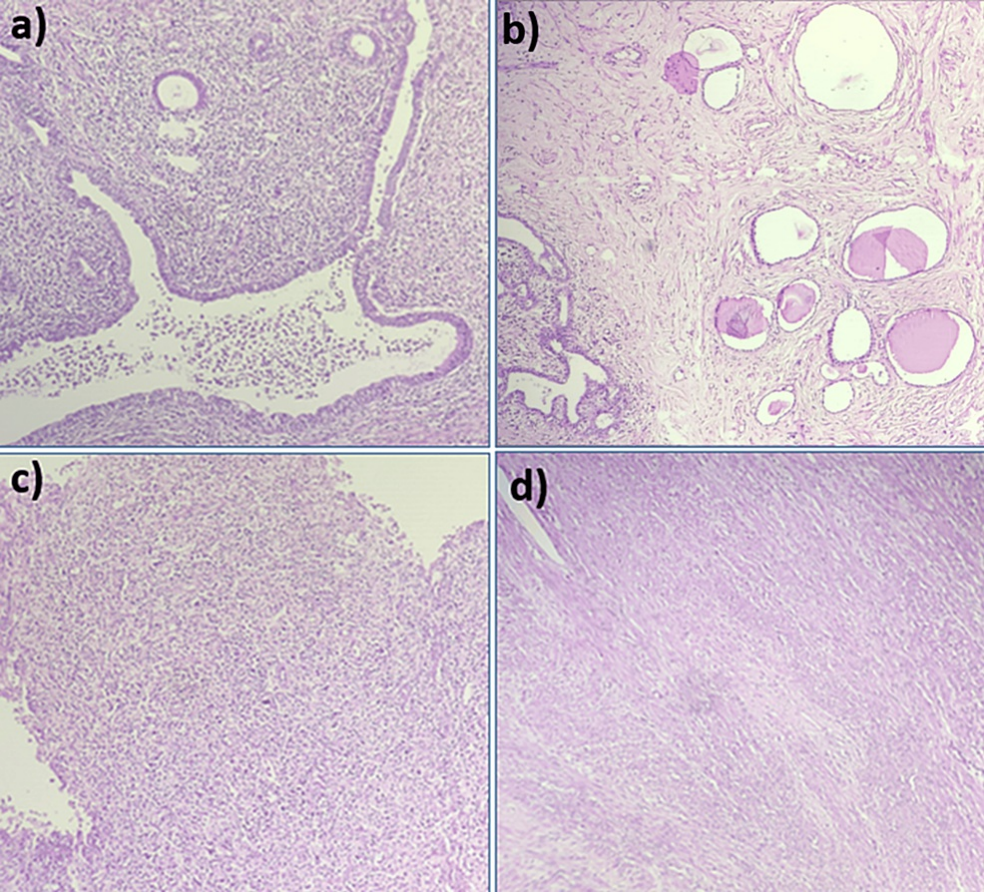
Histopathological imaging (a) Biphasic tumor with cysts lined by columnar epithelium and stroma composed of fascicles of spindle cells. (b) Tubules with thyroidization. (c) A focus showing chronic inflammation with giant cells. (d) Mesenchymal component predominantly showing smooth muscle differentiation.

Microscopic examination revealed a biphasic neoplasm consisting of epithelial and stromal elements. The epithelial component comprised cysts and glands of varying sizes, lined by columnar cells surrounded by a dense and collagenous stroma composed of spindle-shaped cells. Smooth muscle differentiation areas, along with dense collections of foamy histiocytes, plasma cells, and lymphocytes, were noted. The interstitium displayed scattered tubules with thyroidization (Figure 2).

**Figure 3:**
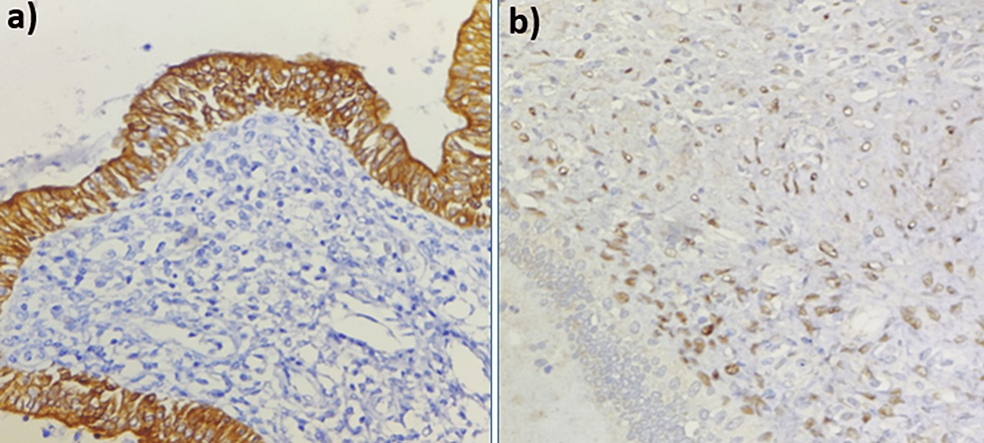
Immunohistochemical analysis (a) Cytokeratin 7 (CK7)-positive epithelial component. (b) Estrogen-positive stromal component.

Immunohistochemistry demonstrated positivity for the estrogen receptor (ER) in stromal cells and for cytokeratin 7 (CK7) in epithelial cells (Figure 3). The pathological and immunohistochemical findings were consistent with the diagnosis of MEST.

## Discussion

MEST is a rare biphasic tumor in adults comprised of both stromal and epithelial elements [1]. It has previously been referred to as cystic nephroma with ovarian or cellular-type stroma and adult mesoblastic nephroma in the literature [8]. While cystic nephroma is recognized as a distinct entity in the WHO classification of renal neoplasms, MEST is characterized by a diverse composition of microcysts, cysts, and tubules with varied cellular-type stroma. Typically observed in perimenopausal and postmenopausal women, especially those with a history of estrogen exposure or long-term hormone replacement therapy, MEST has also been associated with hormone treatments, like leuprolide and diethylstilbestrol, in men [9]. The gender ratio of 6:1 shows that females are more commonly affected, with a mean age at presentation being 46 years [10]. While recurrence, metastasis, and malignant transformations are rare, rare cases of malignant MEST have been reported [4]. 

Patients with MEST typically present with urinary symptoms, such as hematuria, abdominal pain, a palpable mass on the flank, and recurrent urinary tract infections. Interestingly, around 25% of patients may be asymptomatic, and the tumor is often incidentally discovered during abdominal imaging [9]. Radiological investigations are usually inconclusive while distinguishing MEST from other complex renal cysts and cystic nephromas due to their gross morphological similarities. The use of fine-needle aspiration (FNA) in cystic lesions of the kidney is a subject of debate, primarily because of the potential risk of spreading malignant cells, particularly in cases where a cystic renal cell carcinoma is suspected. Ultimately, the conclusive diagnosis relies on the histological examination of the tissue.

In the majority of cases, the gross examination of the tumor reveals a variably shaped, encapsulated tan-yellow area with a mixture of cystic and solid sections, with a predominance of either component. Microscopically, the tumor exhibits both stromal and epithelial components, with the epithelial component not only present on the tumor's surface but also within the mesenchymal tissue. Notably, MEST is not associated with features like necrosis, dysplasia, cellular atypia, enhanced mitotic activity, or other malignant characteristics.

Immunohistochemical staining shows that approximately 90% of spindle-shaped stromal cells are positive for smooth muscle actin, desmin, estrogen receptor (ER), and progesterone receptor (PR). About 50% of cases are positive for CD10, CD34, and WT1. The epithelial component stains positive for PAX8 and GATA3, while the tumor cells are negative for inhibin, SF1, HMB45, and cathepsin. Despite its excellent prognosis, total surgical excision is the preferred treatment for MEST. However, additional testing is often required to rule out malignant transformation [5].

It is noteworthy that cystic nephroma is a differential diagnosis for tumors that may present as MEST, as they share several clinical, morphological, histopathological, and immunohistochemical features. Turbiner et al. proposed combining them into a single entity called "renal epithelial and stromal tumors" (RESTs) [8].

## Conclusions

When a perimenopausal woman presents with a renal mass, it is important to consider MESTs of the kidney as a potential diagnosis, especially since it represents a distinct entity among benign kidney tumors. While rare cases of malignant transformation have been reported, the majority of MEST lesions are benign. The diagnosis should be approached by considering the patient's age and histopathological features, as MEST typically has a favorable prognosis, and conservative surgery is often effective in its management.
